# Supplementary material for: Identification of photoperiod-induced specific miRNAs in the adrenal glands of Sunite sheep (Ovis aries)
Source: Front Vet Sci. 2022 Jul 22;9:888207. doi: 10.3389/fvets.2022.888207 (PMC9354845; doi:10.3389/fvets.2022.888207)
Supplement: Supplementary file 1 [file Data_Sheet_1.ZIP › Supplementary file/miRDeep2 file.zip!/miRDeep2 file/Know_Structure/SP42A1/oar-mir-30c.pdf]

miRBase precursor : oar-mir-30c
Total read count : 73090
oar-miR-30c read count : 72948
remaining reads : 142

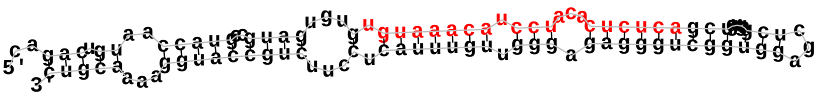

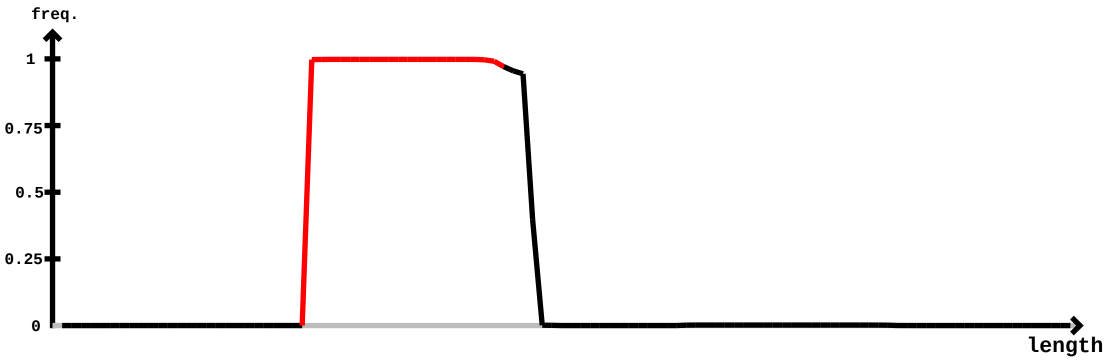

oar-miR-30c

5'- cagacuguaaccaugccguagugugUGUAAACAUCCUACACUCUCAgcugugagcucgagguggcugggagaggguuguuuacuccuucugccauggaaaacguc -3' exp

..(((.((..(((((..((((...(.(((((((.(((...(((((((((....(((...)))))))))))).))).))))))).)...)))))))))...)))))	reads	mm	sample
cagacuguaaccaugccguagugu...	1	0	seq
.agacuguaaccaugccguagugug..	1	0	seq
....cuguaaccaugccguagugug..	2	0	seq
.........................uguaaacauccuacacu...	1	0	seq
.........................uguaaacauccuaUacu...	1	1	seq
.........................uguaaacauccuacacuc..	79	0	seq
.........................uguaaacauccuacacAc..	1	1	seq
.........................uguaaacauccuacacuU..	36	1	seq
.........................uguGaacauccuacacuc..	2	1	seq
.........................uguaaacauccuacacucu...	371	0	seq
.........................uguaaacauccuacacuUu...	1	1	seq
.........................uguGaacauccuacacucu...	1	1	seq
.........................uguaaacaCccuacacucu...	25	1	seq
.........................uguaaacauccuacacucuc..	1382	0	seq
.........................uguaaacaCccuacacucuc..	109	1	seq
.........................uguaaacauccuacacucuca...	1059	0	seq
.........................uguaaacaCccuacacucuca...	94	1	seq
.........................uguaaacauccuacacucucag..	715	0	seq
.........................uguaaacaCccuacacucucag..	79	1	seq
.........................uguaaacauccuacacucucagc...	37103	0	seq
.........................uguaaacaCccuacacucucagc...	2835	1	seq
.........................uguaaacaCccuacacucucagcu..	1689	1	seq
.........................uguaaacauccuacacucucagcu..	27214	0	seq
.........................uguaaacauccuacacucucagcug...	22	0	seq
.........................uguaaacaCccuacacucucagcug...	3	1	seq
.........................uguaaacauccuacacucucagcugu..	68	0	seq
..........................guaaacauccuacacucuc..	1	0	seq
..........................guaaacaCccuacacucuca...	1	1	seq
..........................guaaacauccuacacucuca...	1	0	seq
..........................guaaacauccuacacucucagc...	14	0	seq
..........................guaaacaCccuacacucucagc...	2	1	seq
..........................guaaacaCccuacacucucagcu..	2	1	seq
..........................guaaacauccuacacucucagcu..	21	0	seq
...........................uaaacauccuacacucucagc...	12	0	seq
...........................uaaacauccuacacucucagcu..	3	0	seq

oar-miR-30c

```
cagacuguaaccaugccguagugug uguaaacauccuacacucuca gcugugagcucgagguggcugggagaggguuguuuacuccuucugccauggaaaacguc
...........................uaaacaCccuacacucucagcu........................................................ 1 1 seq
................................................................cugggagaggguuguuua....................... 1 0 seq
................................................................cugggagaggguuguuuacu..................... 9 0 seq
................................................................cugggagaggguuguuuacuc.................... 23 0 seq
................................................................cugggagaggguuguuuacucc................... 77 0 seq
................................................................cugggagaggguuguuuacuccu.................. 5 0 seq
................................................................cugggagaggguuguuuacuccuu................. 4 0 seq
.................................................................ugggagaggguuguuuacu..................... 2 0 seq
.................................................................ugggagaggguuguuuacuc.................... 2 0 seq
.................................................................ugggagaggguuguuuacucc................... 13 0 seq
.................................................................ugggagaggguuguuuacuccuu................. 2 0 seq
```
